# COVID-19 and cancer patients

**DOI:** 10.1590/0100-3984.2020.53.4e1

**Published:** 2020

**Authors:** Dante Luiz Escuissato

**Affiliations:** 1 Associate Professor of Radiology at Universidade Federal do Paraná (UFPR), Curitiba, PR, Brazil. Email: dante.escuissato@gmail.com.

Coronavirus disease 2019 (COVID-19) started in the city of Wuhan, China, and quickly spread around the world, becoming a pandemic. It continues to be a serious public health problem.

On computed tomography (CT), pulmonary infection with severe acute respiratory syndrome coronavirus 2 (SARS-CoV-2) is characterized by ground-glass opacities and consolidations, with a bilateral distribution. Cavitary lung lesions, small pulmonary nodules, and pleural effusion are atypical; when any of those are identified, other causes should be investigated^(1,2)^.

The sensitivity and specificity of chest CT vary widely (60-98% and 25-53%, respectively). The positive predictive value (PPV) and negative predictive value (NPV) are estimated at 92% and 42%, respectively. Due to the relatively low NPV, the Brazilian College of Radiology and Diagnostic Imaging, as well as several international entities, recommend that chest CT not be used in the screening for COVID-19. However, in a clinical context, its use is justified when diagnostic resources such as reverse-transcriptase polymerase chain reaction and serological tests are not available^(3,4)^.

More than 18 million new cases of cancer appear each year worldwide. Because of the coexistence of chronic diseases and poor general health status, together with the immunosuppression caused by the neoplasia and the treatment regimens, individuals with cancer are more susceptible to infection. As a consequence, cancer patients infected with SARS-CoV-2, especially those with hematologic malignancies, lung cancer, or advanced metastatic disease, may have a worse evolution and prognosis when compared with other populations of SARS-CoV-2-infected individuals^(5)^.

Zhang et al.^(6)^ observed that the chest CT abnormalities seen in cancer patients with COVID-19 pneumonia were similar to those seen in the general population of patients with pneumonia. The findings included ground-glass opacities (in 75.0%), multifocal consolidations (in 46.3%), and bilateral lesions (in 78.6%). The authors suggested that the worse prognosis in some cancer patients may be related to delayed hospitalization.

In this issue of **Radiologia Brasileira**, Barbosa et al.^(7)^ presented a study conducted at a cancer center in Brazil where two different approaches were taken to determine whether chest CT findings should be considered positive for COVID-19. The authors observed that, in scenario 1 (in which positivity was defined on the basis of typical chest CT findings alone), sensitivity was lower, whereas specificity and NPV were higher, than what is found in the medical literature. In scenario 2 (in which positivity was defined on the basis of typical and indeterminate chest CT findings), the accuracy was similar to that reported in other studies. In both scenarios, the NPV was higher than that reported elsewhere. These differences reflect the current state of knowledge of this virus, which has various clinical and epidemiological characteristics, raising new questions and calling for ongoing review of concepts.
